# Identification of Iminium Intermediates Generation in the Metabolism of Tepotinib Using LC-MS/MS: In Silico and Practical Approaches to Bioactivation Pathway Elucidation

**DOI:** 10.3390/molecules25215004

**Published:** 2020-10-28

**Authors:** Ali S. Abdelhameed, Mohamed W. Attwa, Adnan A. Kadi

**Affiliations:** Department of Pharmaceutical Chemistry, College of Pharmacy, King Saud University, P.O. Box 2457, Riyadh 11451, Saudi Arabia; mzeidan@ksu.edu.sa (M.W.A.); akadi@ksu.edu.sa (A.A.K.)

**Keywords:** tepotinib, in vitro phase I metabolites, DEREK module of StarDrop software, human liver microsomes, iminium intermediates

## Abstract

Tepotinib (Tepmetko™, Merck) is a potent inhibitor of c-Met (mesenchymal−epithelial transition factor). In March 2020, tepotinib (TEP) was approved for use in Japan for the treatment of patients who suffered from non-small cell lung cancers (NSCLC) harboring an MET exon 14 skipping alteration and have progressed after platinum-based therapy. Practical and in silico experiments were used to screen for the metabolic profile and reactive intermediates of TEP. Knowing the bioactive center and structural alerts in the TEP structure helped in making targeted modifications to improve its safety. First, the prediction of metabolism vulnerable sites and reactivity metabolic pathways was performed using the StarDrop WhichP450™ module and the online Xenosite reactivity predictor tool, respectively. Subsequently, in silico data were used as a guide for the in vitro practical work. Second, in vitro phase I metabolites of TEP were generated from human liver microsome (HLM) incubations. Testing for the generation of unstable reactive intermediates was performed using potassium cyanide as a capturing agent forming stable cyano adduct that can be characterized and identified using liquid chromatography tandem mass spectrometry (LC-MS/MS). Third, in silico toxicity assessment of TEP metabolites was performed, and structural modification was proposed to decrease their side effects and to validate the proposed bioactivation pathway using the DEREK software. Four TEP phase I metabolites and four cyano adducts were characterized. The reactive intermediate generation mechanism of TEP may provide an explanation of its adverse reactions. The piperidine ring is considered a structural alert for toxicity as proposed by the DEREK software and a Xenosite reactivity model, which was confirmed by practical experiments. Steric hindrance or isosteric replacement at α-carbon of the piperidine ring stop the bioactivation sequence that was confirmed using the DEREK software. More drug discovery studies can be performed using this perception permitting the design of new drugs with an increased safety profile. To our knowledge, this is the first study for the identification of in vitro phase I metabolites and reactive intermediates in addition to toxicological properties of the metabolites for TEP that will be helpful for the evaluation of TEP side effects and drug–drug interactions in TEP-treated patients.

## 1. Introduction

Tepotinib (TEP) is a potent inhibitor of c-Met (mesenchymal−epithelial transition factor). On 11 September 2019, Merck KGaA (Darmstadt, Germany) announced that the FDA had granted a breakthrough therapy designation to TEP (Tepmetko™) for the management of patients with non-small cell lung cancers (NSCLC) harboring an MET exon 14 skipping alteration who have progressed subsequent platinum-based therapy [1]. The designation is based on data from the current VISION study, which revealed promising clinical evidence for TEP in metastatic NSCLC harboring METex14 skipping alterations [2]. Two MET inhibitors (TEP and Capmatinib) received a breakthrough therapy designation from the FDA for the treatment of NSCLC patients who have MET exon 14 skipping mutations [3]. In the third quarter of 2019, TEP was granted orphan drug designation by the Japanese Ministry of Health, Labour and Welfare for use in Japan in this indication, and in March 2020 was approved [4]. The most common side effects of TEP were nausea (23.0%), peripheral edema (48.3%), blood creatinine increase (12.6%) and diarrhea (20.7%). Grade 3 peripheral edema was seen in 8% of patients. Some patients stopped treatment due to side effects (peripheral edema, interstitial lung disease, diarrhea and nausea) [2].

Reactive metabolites are considered electron deficient systems and thus can modify DNA and proteins through the creation of covalent bonds with the active sites, which is considered the first step in organ toxicities that were mediated by drugs [5,6,7]. Detection of reactive metabolite generation in drug metabolism of new molecule is a significant job in explaining drug-induced toxicity [8,9,10,11]. Reactive metabolites are often generated in the phase I metabolism stage and cannot be characterized directly due to its instability. So, a capturing nucleophile can be used to interact with the bioactive metabolites forming stable adducts, which can be separated and identified by liquid chromatography tandem mass spectrometry (LC-MS/MS) [12,13]. TEP chemical structure involves piperidine (cyclic tertiary amine moiety). The piperidine ring can go through bioactivation by iminium ion formation that can be trapped using KCN [14,15,16,17]. The adducts that are generated through electrophilic-nucleophilic reaction are considered stable and can be separated and identified by LC-MS/MS [12,13,14,18,19]. We proposed that these reactive metabolites are participated in the TEP reported toxic side effects. Although the metabolism of TEP was studied before, the cyano adducts were not detected in the previous published work as expected from in silico and practical experiments for similar drugs containing the piperidine ring [20,21,22,23]. This encouraged us to carry out more in silico and practical work to explain the role of piperidine group on the bioactivation of TEP and its side effects.

StarDrop software package uses a quantum mechanical approach for the proposal of the relative involvement of different metabolic enzymes (CYP3A4, 2D6 and 2C9) of the tested compound. Its mechanism depends on the computation of the energy barrier to the electron removal that is represented by the rate-limiting step in product generation [24]. The Derek module is involved in the StarDrop package software. It is a knowledge-based expert system for the prediction of toxicity [25]. The Derek software is composed of alerts, examples and rules that may each contribute to the toxicity predictions made by the system. This in silico software was used for predictions of metabolites and its toxicological side effects. The Xenosite reactivity model is an online website that accepts input in common chemical file formats including SMILES and SDF [26,27]. The vulnerable electrophilic atomic sites of the studied drug were proposed for DNA, cyanide, GSH and protein. The outcomes were represented according to a color scale bar that has a color gradient. The in silico data were used to guide the practical experiments.

In the current study, we isolated and characterized in vitro phase I and reactive metabolites of TEP that were formed in HLM incubation using LC-MS/MS. Checking the generation of reactive intermediates in TEP metabolism using KCN as a trapping agents and the bioactivation pathway was proposed. These data and outcomes could be utilized to facilitate designing new chemical entities with lower side effects maintaining their pharmacological activity [28] through blocking of bioactive sites that are responsible for reactive intermediate formation using isosteric replacement or steric hindrance strategies. In silico toxicity (DEREK software) was used to approve the bioactivation theory and toxicity assessment for TEP metabolites. Structural modifications in the chemical structure of TEP were supposed to eliminate their side effects [26,27,29].

## 2. Results and Discussion

### 2.1. Results of In Silico TEP Metabolites Prediction

**Figure 1:**
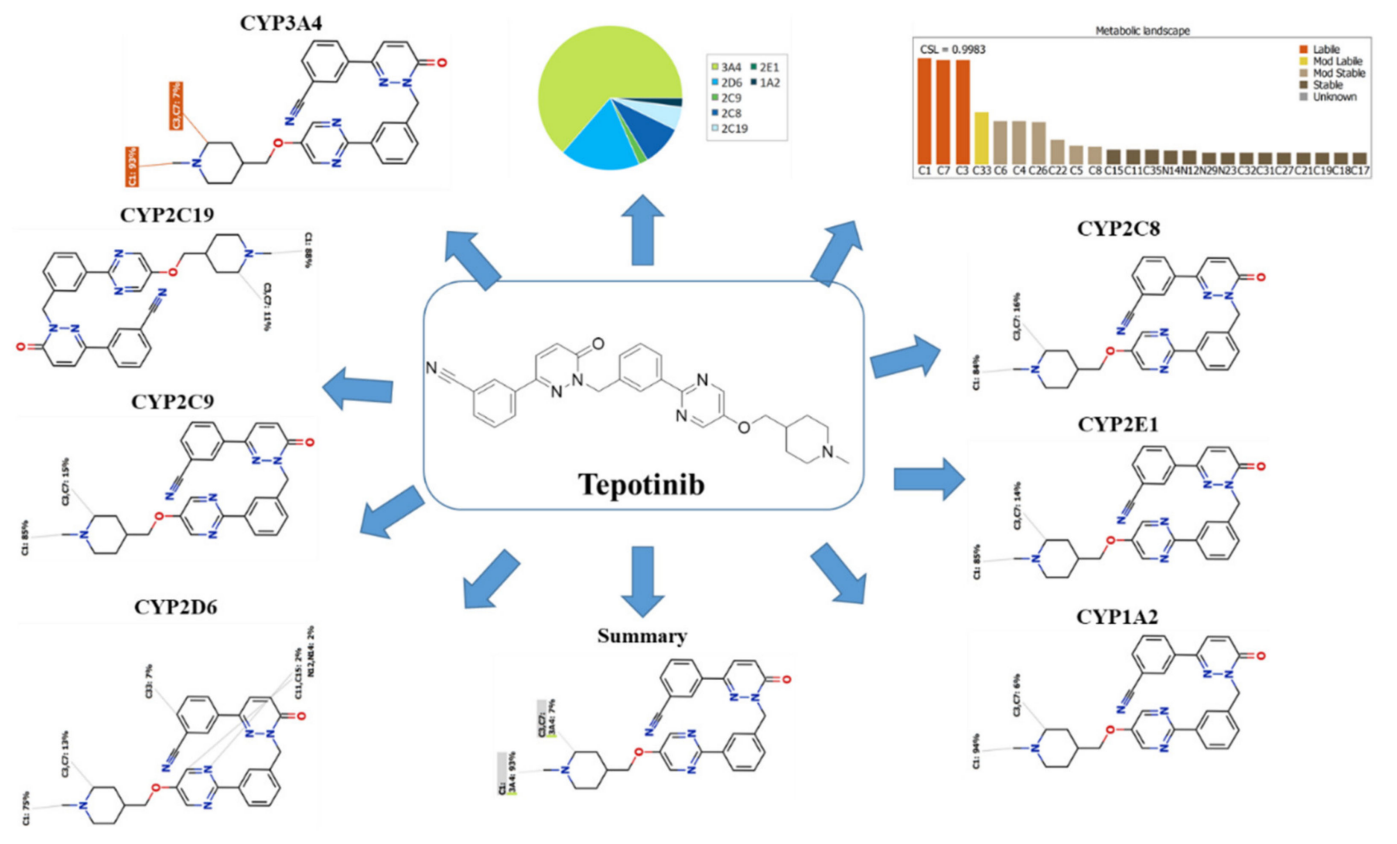
Predicted metabolic sites for tepotinib (TEP) by WhichP450™ module of StarDrop software.

The TEP Metabolic Landscape indicates the lability of each site with respect to metabolism by CYP3A4 in absolute terms to guide TEP metabolites prediction and also chemical structure optimization for enhancing metabolic stability. This indicates that the *N*-methyl piperidine ring is proposed to vulnerable sites for metabolism, which matched with practical work as all metabolic pathways occurred at the piperidine ring (M1 to M4). The composite site liability (CSL) is displayed in the top-right side of the metabolic landscape. The CSL was 0.9983, which is considered high compared to other previously studied anti-cancer drugs (ex. Talazoparib) [30]. The outcomes from the WhichP450™ module of StarDrop software revealed that CYP3A4 was found to have a basic role in TEP metabolism, which proposed the high possibility of drug–drug interaction with other drugs that induce or inhibit CYP3A4 enzyme. Other cytochrome P450 enzymes play a minor role in TEP metabolism (Figure 1).

### 2.2. Results of In Silico TEP Structural Alerts Sites and Toxicity Prediction

**Figure 2:**
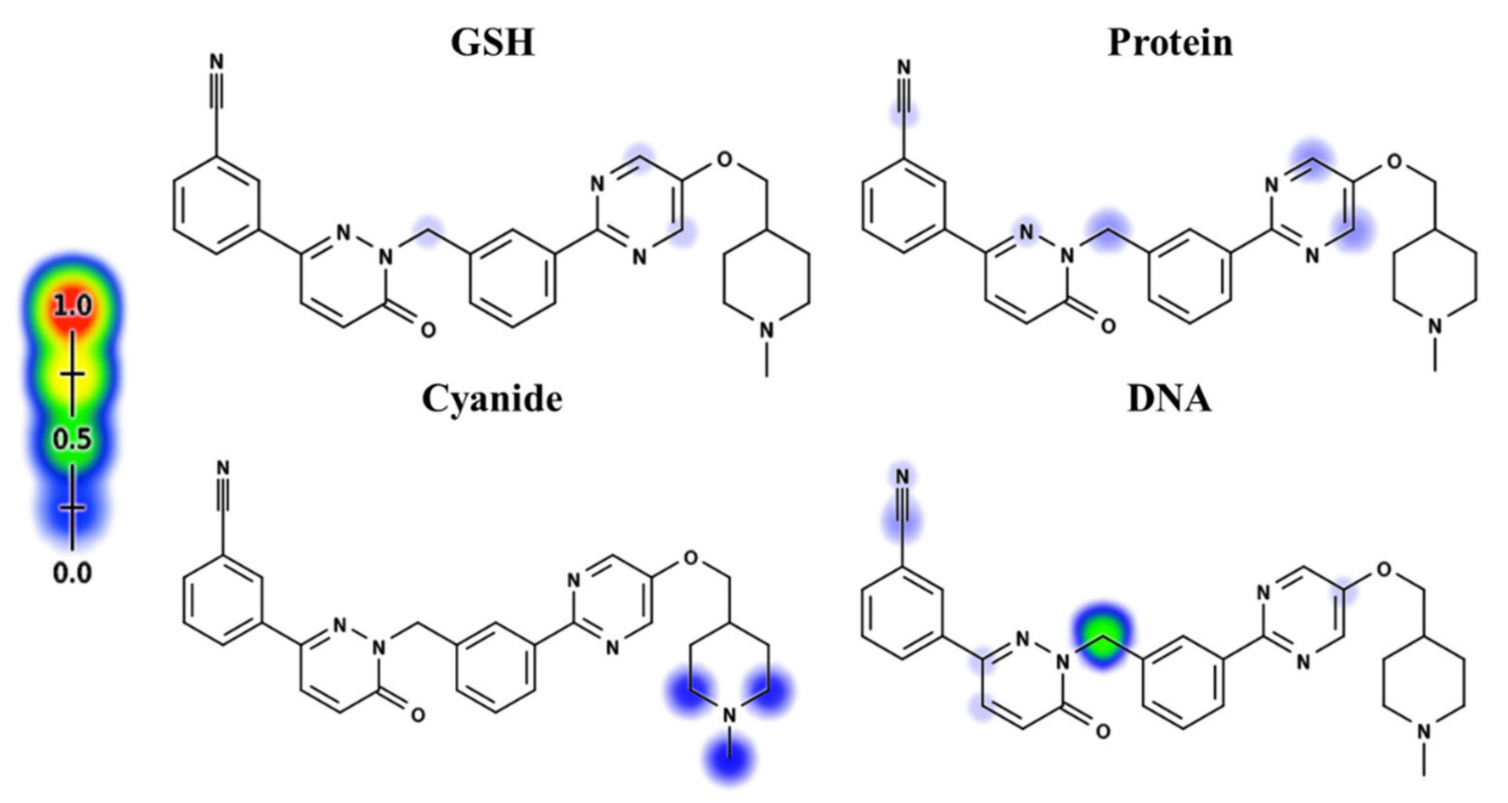
Xenosite reactivity model for TEP including GSH, protein, DNA and cyanide models. Red color indicates the highest scale for probability.

Depending on in silico outcomes and the literature knowledge, predicted metabolites and reactive intermediates were listed. The vulnerable electrophilic atomic sites of TEP were proposed for DNA, cyanide, GSH and protein by an online Xenosite reactivity model. The outcomes were represented according to a color scale bar that has a color gradient. The red color indicates the maximum possibility of reactive intermediate generation at this site, while the white color indicates no possibility of reactive intermediate formation at the piperidine ring (Figure 2). The DNA model proposed high probability of reactive intermediate formation as indicated by a higher scale. The KCN model indicates that the piperazine ring cyano adduct formation is likely to generate a reactive iminium ion intermediate.

**Figure 3:**
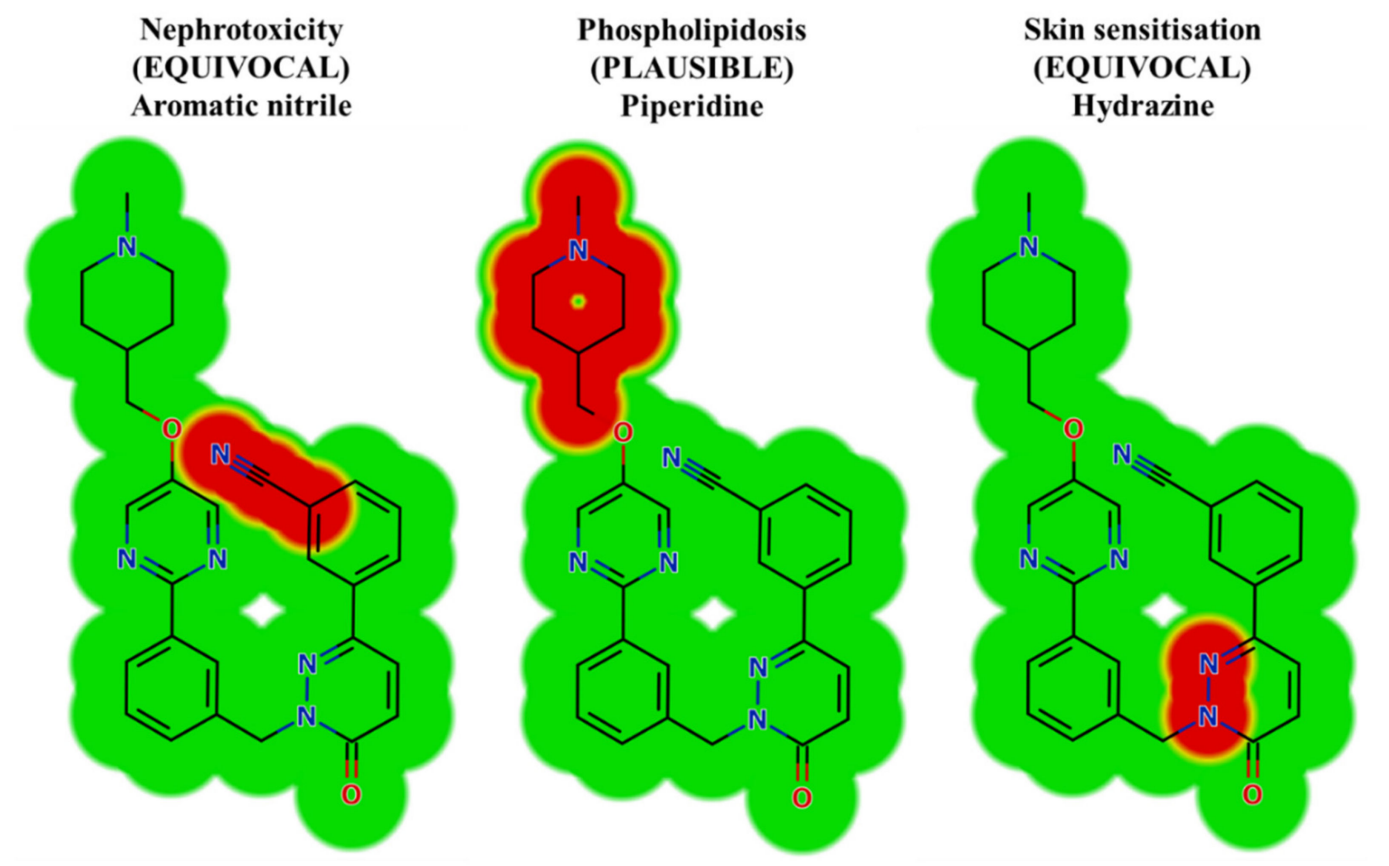
Results of TEP toxicity by DEREK module of StarDrop software exhibiting structural alerts with the predicted side effects of TEP. Red color reveals the structural alerts.

In silico toxicity evaluation of TEP and its phase I metabolites was performed by utilizing the DEREK module of StarDrop software revealing structural alerts (Figure 3). TEP and its metabolites show nephrotoxicity (equivocal), phospholipidosis (plausible) and skin sensitization (equivocal) due to aromatic nitrile, piperidine and hydrazine moieties, respectively. This phospholipidosis alert (plausible) describes the potential induction of piperidine [31]. Phospholipidosis is the build-up of excess intracellular phospholipids. Whilst the majority of body tissues can be susceptible, the lung and alveolar macrophages are the most commonly affected that may be responsible for TEP severe side effects (interstitial lung disease, peripheral edema). Table 1 shows a TEP and its metabolites with DEREK outcomes. The outcomes of the predictions are shown in Figure 3.

### 2.3. Identification of In Vitro Phase I TEP Metabolites

Four TEP metabolites were formed in phase I metabolism by three major phase I metabolic reactions (hydroxylation, *N*-demethylation and oxidation). Four iminium intermediates (cyano adducts) were identified (Table 2).

#### 2.3.1. TEP Fragmentation Pattern

**Scheme 1:**
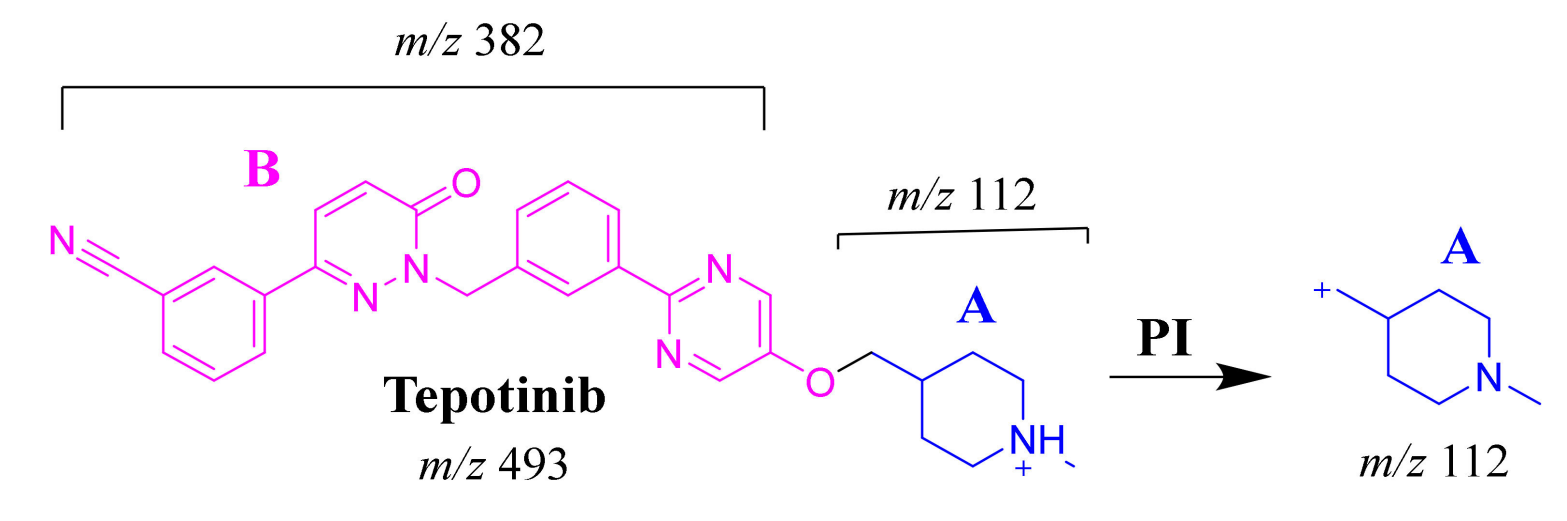
TEP MS/MS fragment ions.

**Figure 4:**
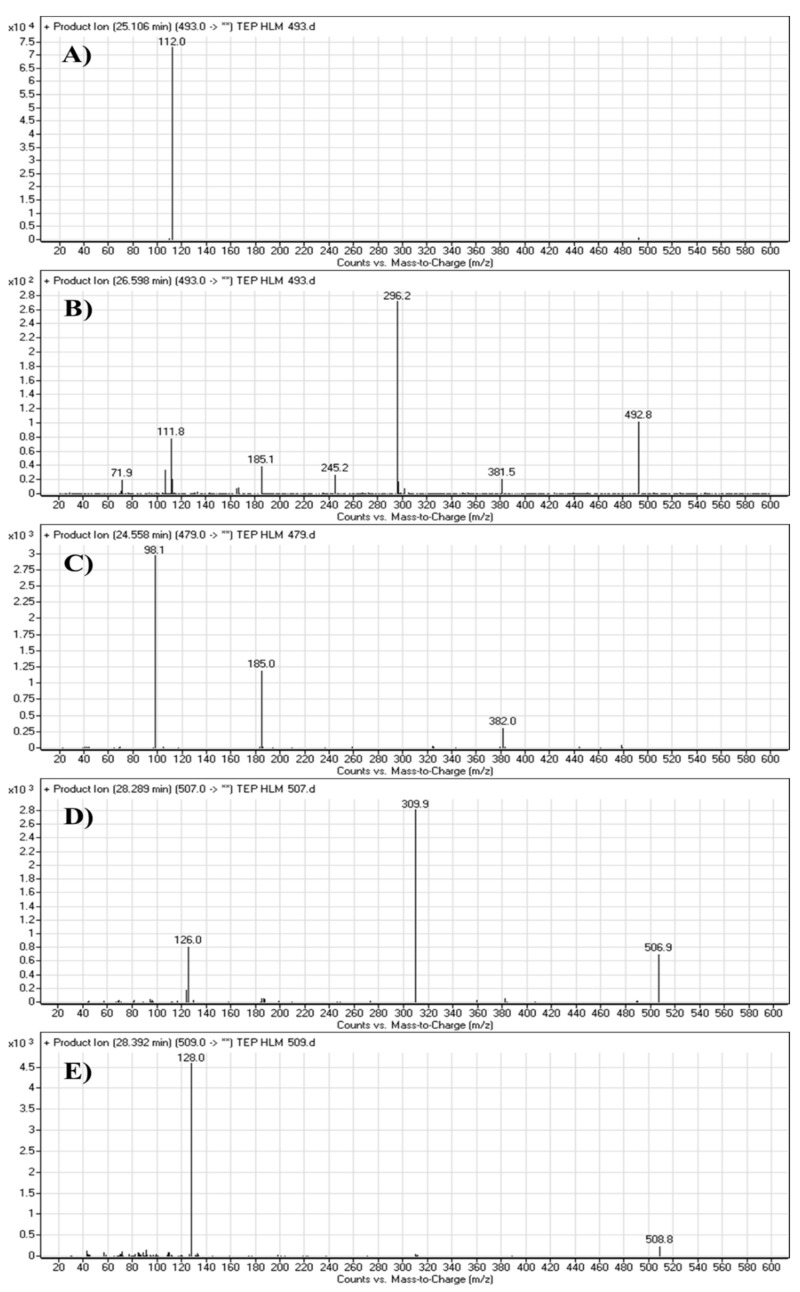
Fragment ions of TEP (**A**). Fragment ions of TEP in vitro metabolites: M1 (**B**), M2 (**C**), M3 (**D**) and M4 (**E**).

The TEP molecular ion peak (MIP) at *m*/*z* 493 was detected at 25.1 min in the total ion chromatogram (TIC). Flow injection analysis was used to adjust the collision energy (CE) for TEP fragmentation. Collision-induced dissociation (CID) of the TEP ion generated only one fragment ion at *m*/*z* 112 whatever the collision energy used, as the methyl piperidine ring bond is considered the weakest point in the structure (Figure 4A). Different CE values were tested (6, 8, 10, 12, 14, 16, 18, 20, 22, 24, 25, 26 and 30). The best collision energy was 25, which gave the highest ion signal (*m/z*: 112). The same CEs were used for all TEP metabolites and adducts. To facilitate localization of metabolic reactions, the chemical structure of TEP was marked as two substructures (A and B). Substructure A (*m*/*z* 112) represents the methyl piperidine moiety, while substructure B (*m*/*z* 382) represents the other part of the chemical structure of TEP (Scheme 1).

#### 2.3.2. M1 Fragment Ions

**Scheme 2:**
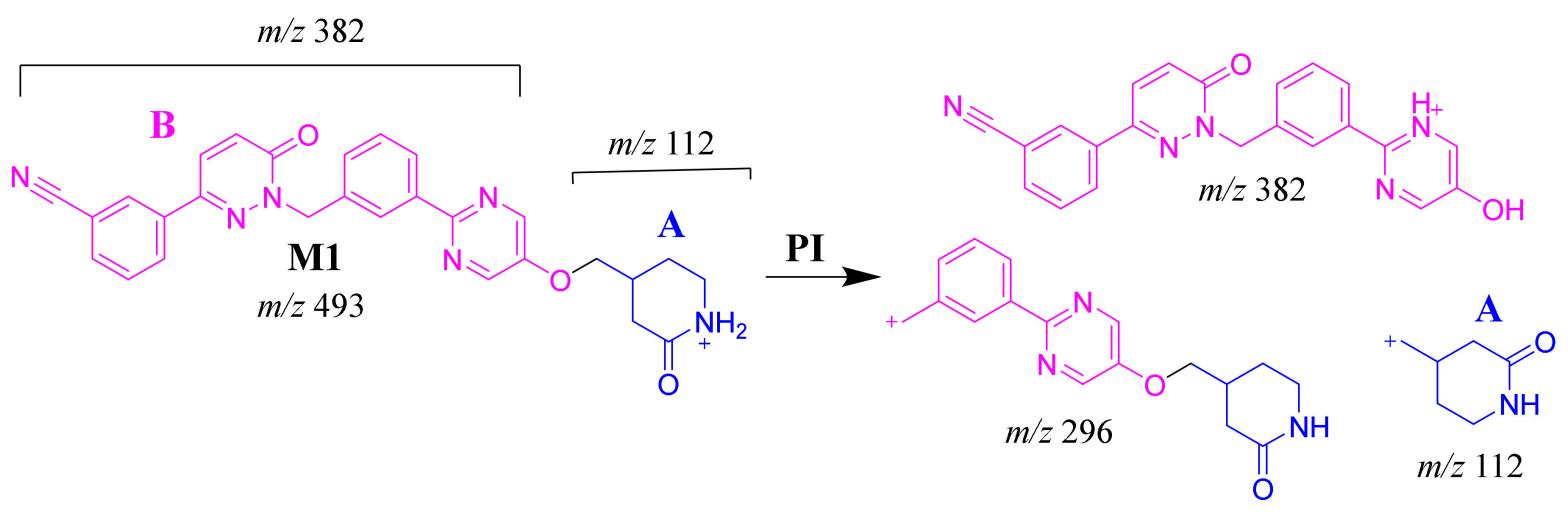
M1 MS/MS fragment ions.

The M1 MIP at *m*/*z* 493 was detected at 26.6 min in the TIC. CID of the M1 ion at *m*/*z* 493 generated five FI at *m*/*z* 382, 296, 185, 112 and 72, which represented fragmentation at soft spots in the chemical structure of TEP493 (Scheme 2). FI at *m*/*z* 382 represents the methyl piperidine ring loss that confirmed no metabolic structure occurred at substructure B. The FI at *m*/*z* 112 proposed the *N*-demethylation and oxidation of α-carbon maintaining the same *m*/*z* of substructure A. The FI at *m*/*z* 72 represent Retro Diels–Alder fragmentation that confirmed oxidation of the piperidine ring. Two other FIs at *m*/*z* 296 and 185 matched with the previous results (Figure 4B).

#### 2.3.3. M2 Fragment Ions

**Scheme 3:**
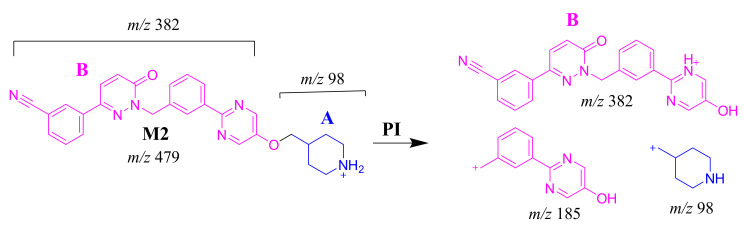
M2 MS/MS fragment ions.

The M2 MIP was detected at 24.6 min in the TIC. CID of the M2 ion at *m*/*z* 479 generated three FIs at *m*/*z* 382, 185 and 98. FI at *m*/*z* 382 represents the loss of the methyl piperidine ring that confirmed no metabolic structure occurred at substructure B (Scheme 3). In comparison to FIs of TEP, the FI at *m*/*z* 98 showed a 14 *m*/*z* decrease, which proposed the *N*-demethylation metabolic reaction of the piperidine ring at substructure A. The FI at *m*/*z* 185 matched with the previous results (Figure 4C).

#### 2.3.4. M3 Fragment Ions

**Scheme 4:**
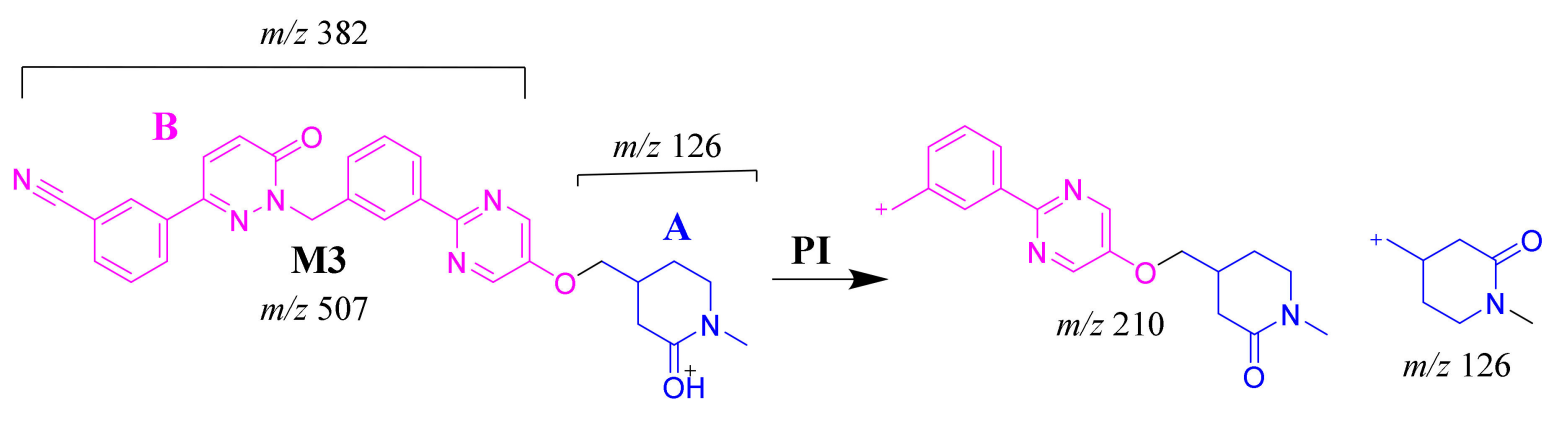
M3 MS/MS fragment ions.

The M3 MIP was detected at 28.3 min in the TIC. CID of the M3 ion at *m*/*z* 507 generated two FIs at *m*/*z* 310 and 126 (Scheme 4). In comparison to FIs of TEP, the FI at *m*/*z* 126 showed a 14 *m*/*z* increase, which proposed the oxidation metabolic reaction of the piperidine ring at substructure A. The FI at *m*/*z* 310 matched with the previous results (Figure 4D).

#### 2.3.5. M4 Fragment Ions

**Scheme 5:**
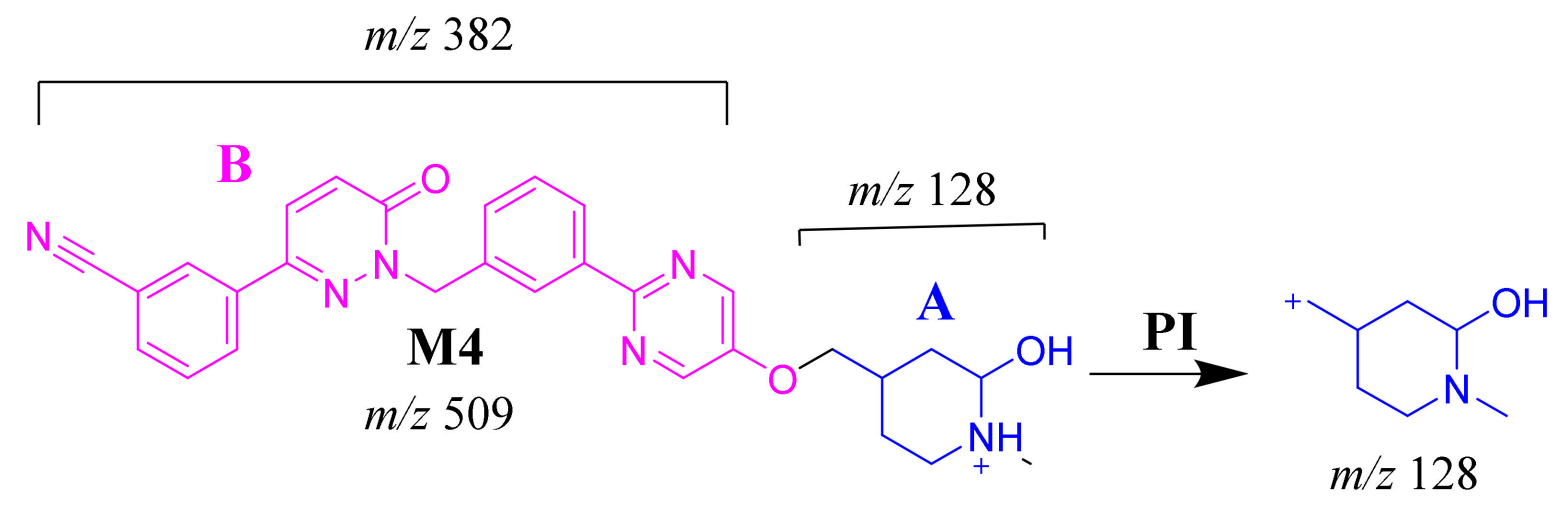
M4 MS/MS fragment ions.

The M4 MIP was detected at 28.4 min in the TIC. CID of the M4 ion at *m*/*z* 509 generated one FI at *m*/*z* 128 (Scheme 5). In comparison to FIs of TEP, the FI at *m*/*z* 128 showed a 16 *m*/*z* increase, which proposed the hydroxylation metabolic reaction of the piperidine ring at substructure A (Figure 4E).

### 2.4. Reactive Metabolites

Four cyano adducts were identified after incubation of TEP with HLMs and KCN as a capturing agent.

#### 2.4.1. TEPCN518 Fragment Ions

**Figure 5:**
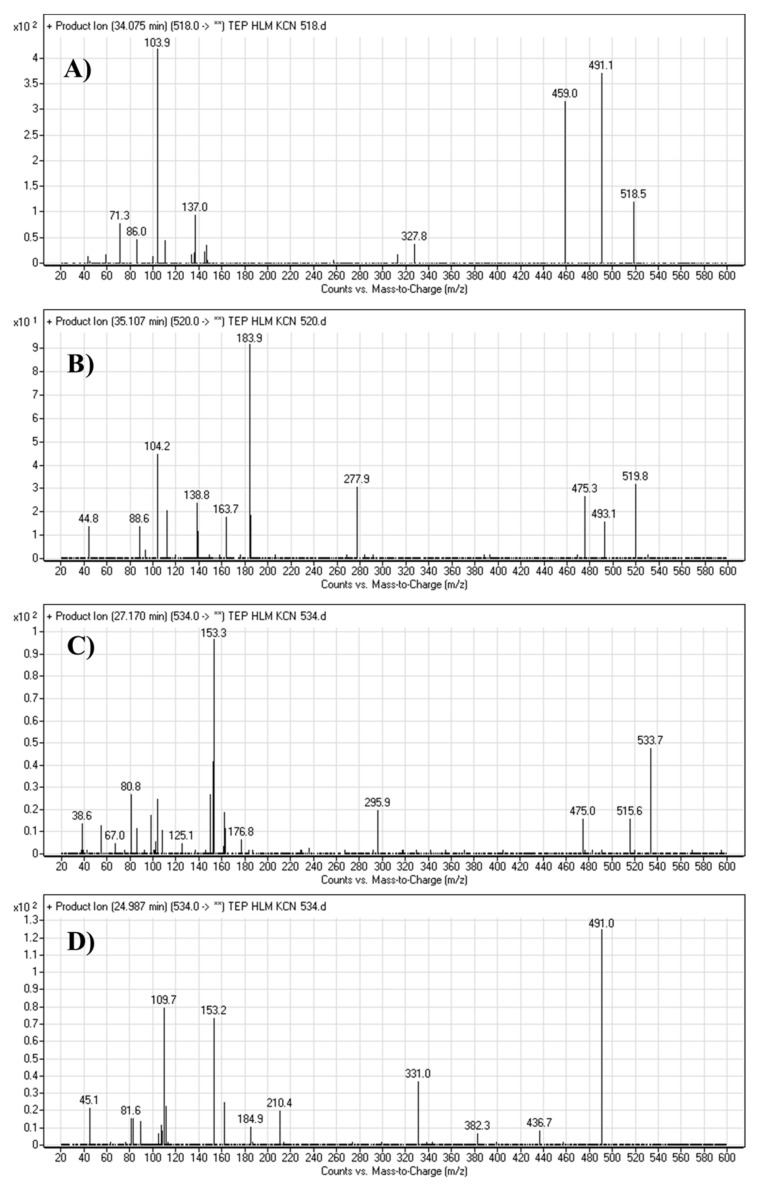
Fragment ions of cyano adducts: TEPCN518 (**A**), TEPCN520 (**B**), TEPCN534a (**C**) and TEPCN534b (**D**) generated in the in vitro metabolism of TEP in the presence of KCN as a trapping agent.

**Scheme 6:**
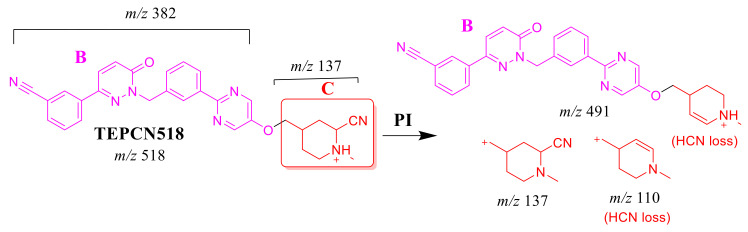
TEPCN518 MS/MS fragments.

The TEPCN518 MIP was detected at 34.1 min in the TIC. CID of the TEPCN518 ion at *m*/*z* 518 generated five qualitative FIs at *m*/*z* 491, 459, 137, 110 and 104 (Scheme 6). In comparison to FIs of TEP, the FI at *m*/*z* 137 showed a 25 *m*/*z* increase, which revealed a cyano attack at the bioactive site of the piperidine moiety (α carbon to tertiary nitrogen atom). The FIs at *m*/*z* 491 and 110 showed a neutral loss of HCN (27 *m*/*z* loss), which is very characteristic of cyano addition. The FIs at *m*/*z* 459 and 104 matched with the previous results (Figure 5A).

#### 2.4.2. TEPCN520 Fragment Ions

**Scheme 7:**
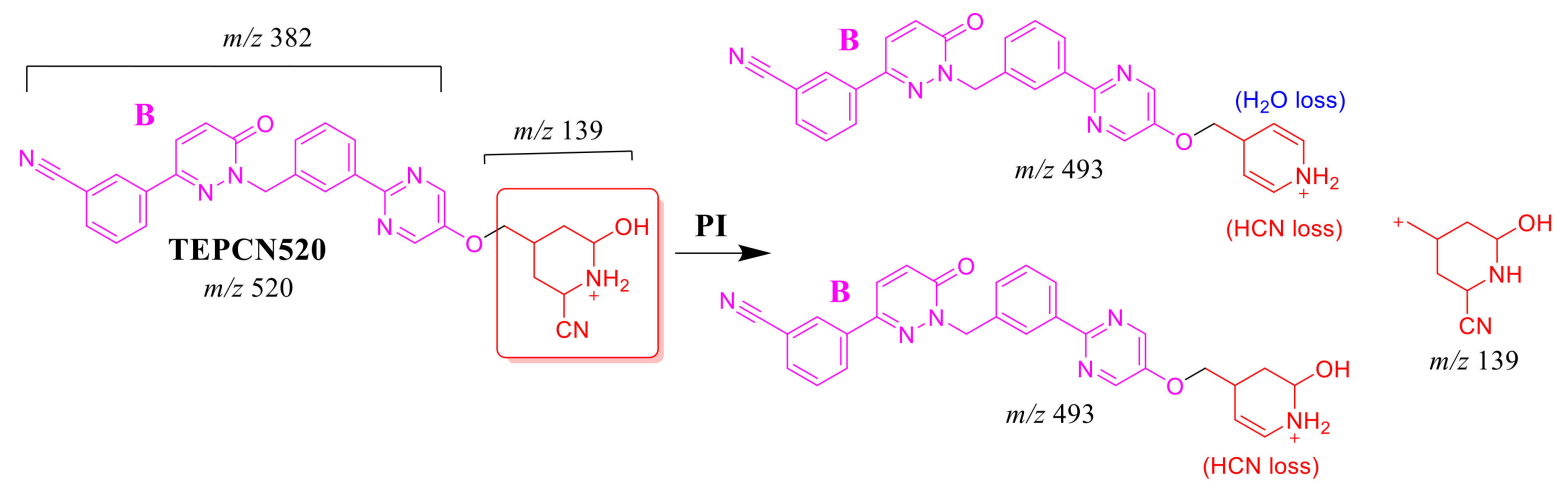
TEPCN520 MS/MS fragments.

The TEPCN520 MIP was detected at 35.1 min in the TIC. CID of the TEPCN520 ion at *m*/*z* 520 generated four characteristic FIs at *m*/*z* 493, 475, 278 and 139 (Scheme 7). In comparison to FIs of TEP, the FI at *m*/*z* 139 exhibited a 27 *m*/*z* increase, which revealed a cyano group addition, *N*-demethylation and hydroxylation of the piperidine ring. The FI at *m*/*z* 493 exhibited a neutral loss of HCN (27 *m*/*z* loss), which is very characteristic of cyano addition. The FI at *m*/*z* 475 revealed a neutral loss of HCN (27 *m*/*z* loss) and water loss, which is very characteristic of cyano addition and hydroxylation metabolic reactions. The FI at *m*/*z* 278 matched with the previous results (Figure 5B).

#### 2.4.3. TEP534a and TEP534b Fragment Ions

**Scheme 8:**
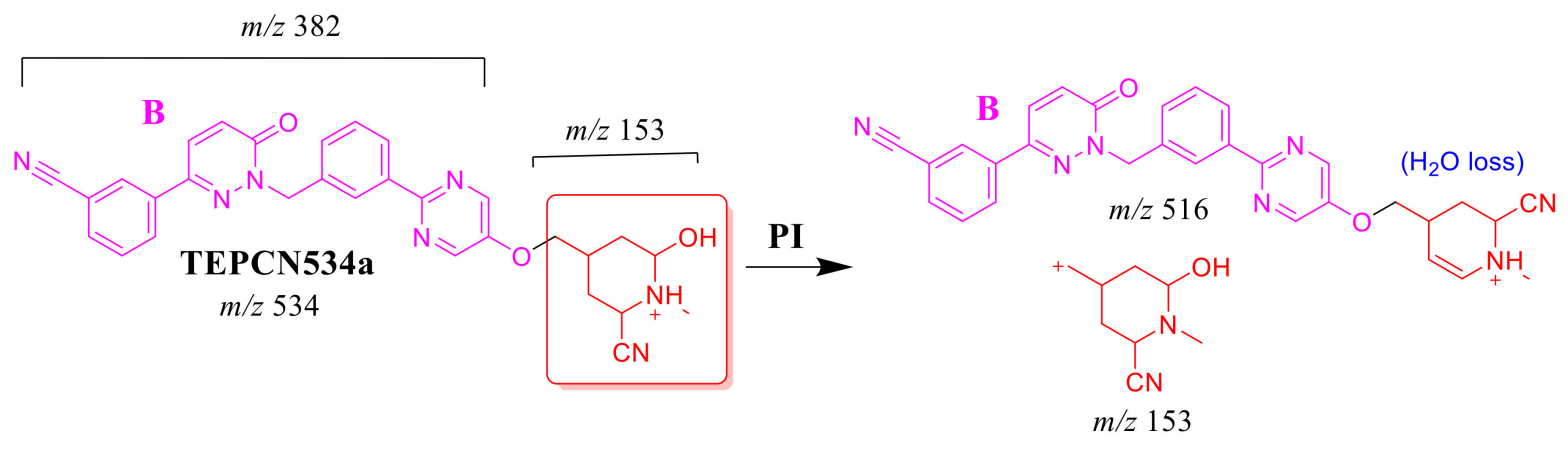
TEPCN534a MS/MS fragments.

The TEP534a and TEP534b MIPs were detected at 27.2 and 24.9 min in the TIC. CID of the ion at *m*/*z* 534 formed different Rt. CID of the TEPCN534a ion at *m*/*z* 534 generated four characteristic FIs at *m*/*z* 516, 475, 296 and 153 (Scheme 8). In comparison to FIs of TEP, the FI at *m*/*z* 153 showed a 41 *m*/*z* increase, which revealed a hydroxylation metabolic reaction and cyano group addition at the piperidine ring of substructure A. The FI at *m*/*z* 516 revealed a neutral loss of H_2_O (loss of 18 *m*/*z* units), which is very characteristic of hydroxylation metabolic reaction. The FI at *m*/*z* 475 and 296 revealed a neutral loss of HCN (27 *m*/*z* loss), which is very characteristic of cyano addition (Figure 5C).

**Scheme 9:**
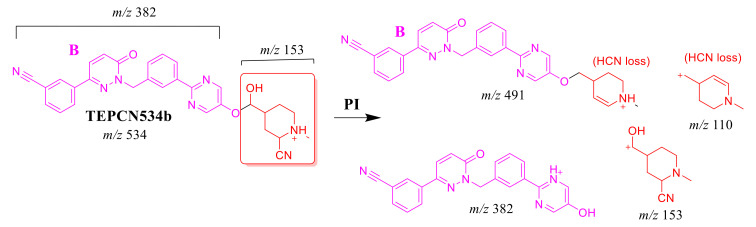
TEPCN534b MS/MS fragments.

CID of the TEPCN534b ion at *m*/*z* 534 generated five characteristic FIs at *m*/*z* 491, 382, 210, 153, and 110 (Scheme 9). In comparison to FIs of TEP, the FI at *m*/*z* 153 showed a 41 *m*/*z* increase, which revealed a hydroxylation metabolic reaction and cyano group addition at the piperidine ring of substructure A that matched with the FIs at *m*/*z* 382 and 210. The FI at *m*/*z* 491 showed a neutral loss of H_2_O (18 *m*/*z* loss) and HCN (27 *m*/*z* loss), which is very characteristic of hydroxylation metabolic reaction and cyano addition, respectively. The hydroxylation metabolic pathway was predicted to occur at the methylene group attached to the piperidine ring, while cyano addition was proposed to occur at the bioactivated α-carbon at the piperidine ring (Figure 5D).

### 2.5. Proposed TEP Bioactivation Mechanism

**Scheme 10:**
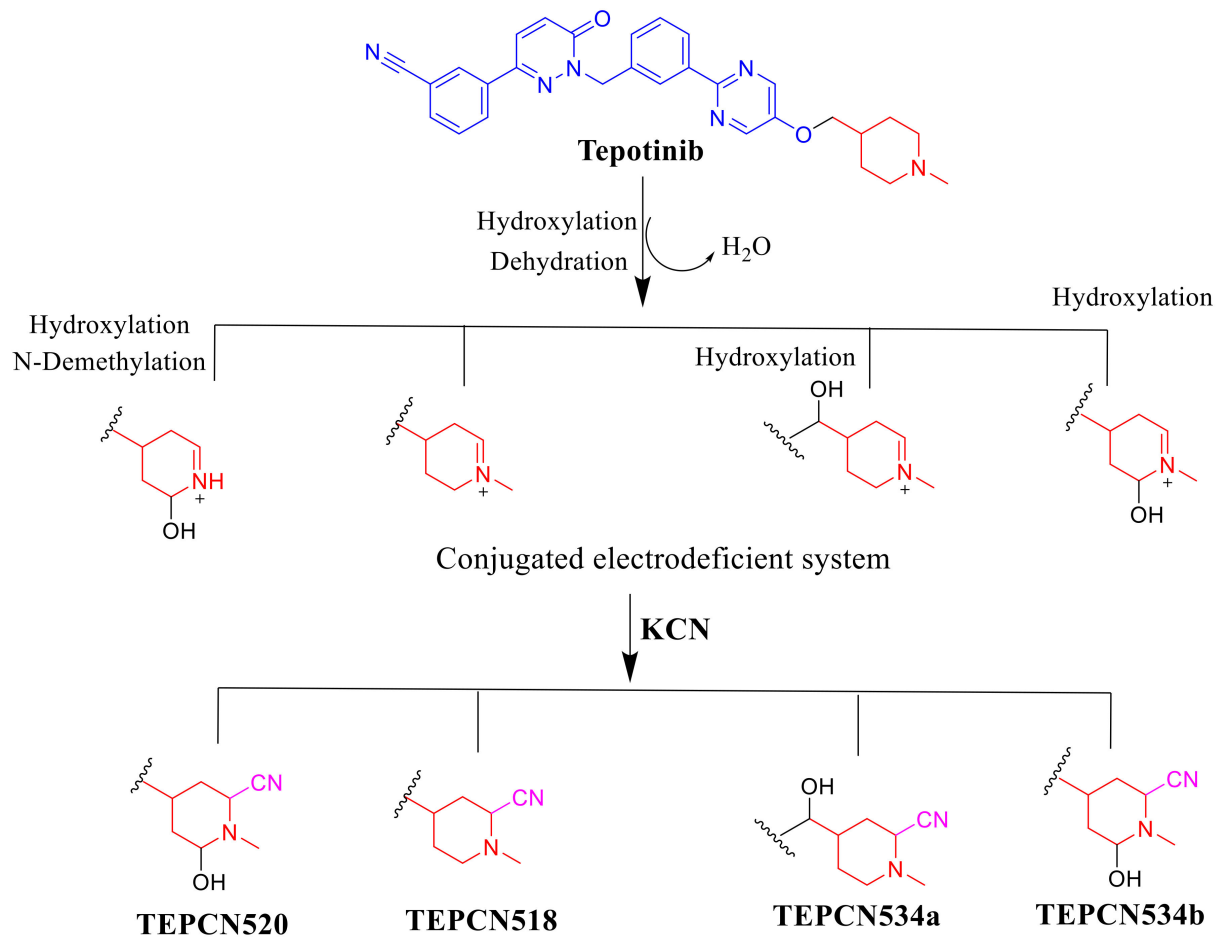
Proposed mechanism of TEP bioactivation.

The generation of four cyano adducts (TEPCN518, TEPCN520, TEPCN534a and TEPCN534b) revealed the formation of unstable reactive intermediates (iminium ions) in TEP metabolism. The bioactivation sequence initiated by hydroxylation metabolic reaction at the bioactivated carbon of the piperidine ring in TEP followed by dehydration (water loss) generated reactive iminium intermediate formation that could be trapped, forming stable cyano adduct (Scheme 10). The predicted bioactivation mechanism of TEP and the iminium intermediate formation have been previously discussed on similar chemical entities that contain cyclic tertiary amine in their chemical structure [32,33].

**Figure 6:**
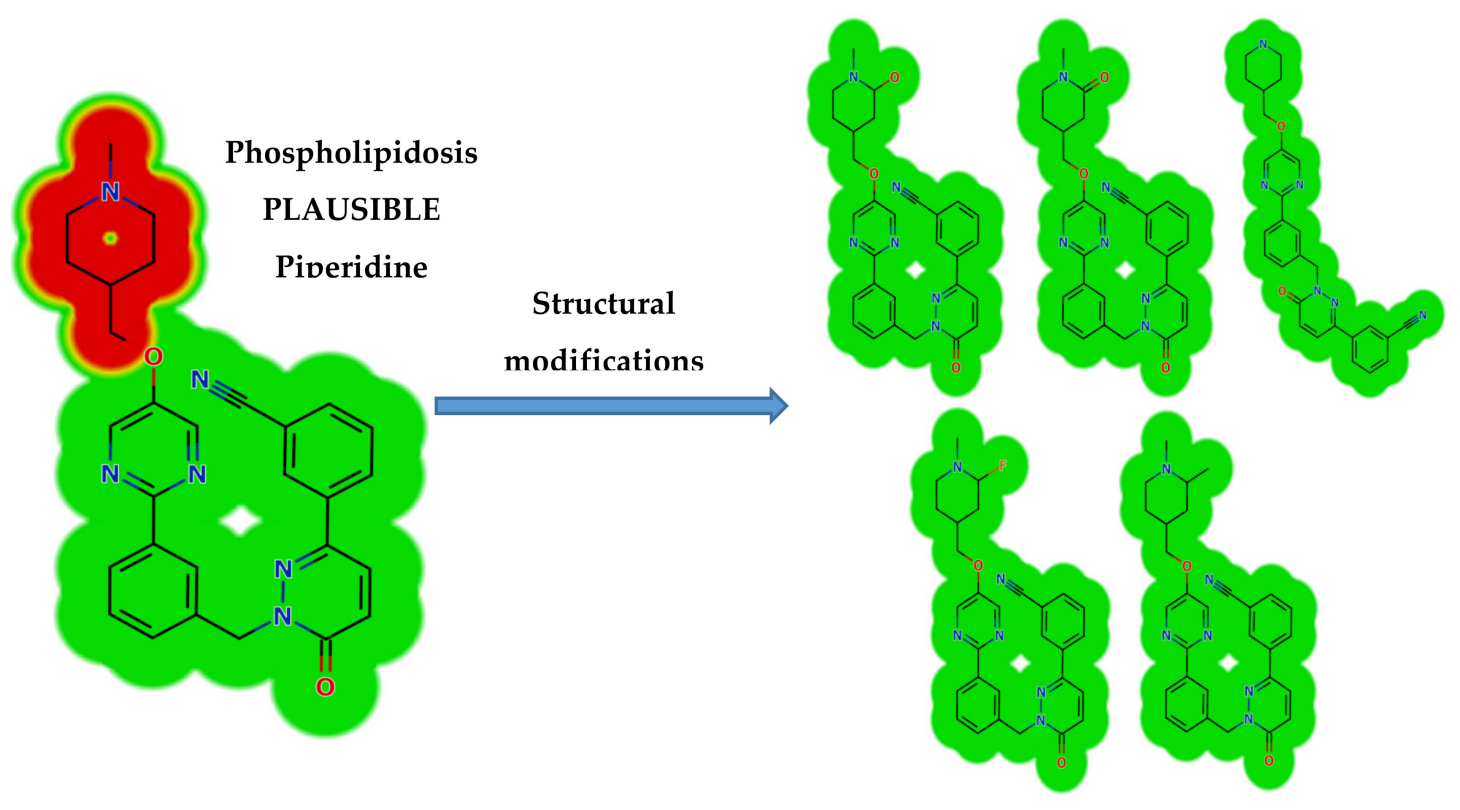
DEREK module of StarDrop software displaying the disappearance of phospholipidosis structural alert due to small structural modification in the *N*-methyl piperidine ring that matched the proposed bioactivation theory.

Structural changes were also proposed to decrease their side effects and to support and confirm the proposed bioactivation pathway theory. Steric hindrance of groups such as methyl or oxygen stops the phospholipidosis toxicity as proposed by DEREK software. Isosteric replacement at α-carbon of the piperidine ring with fluoride atom stops the phospholipidosis toxicity. Additionally, *N*-dealkylation of the tertiary nitrogen of the piperidine ring stops the toxicity. These outcomes matched with the results of the in silico reactivity model and practical bioactivation experiments that approved the role of the piperidine ring in the toxicity of TEP (Figure 6).

## 3. Chemicals and Methods

### 3.1. Chemicals

Pooled HLMs (Product Number: M0567) from various male human livers were purchased from Sigma-Aldrich company (St. Louis, MO, USA) and stored at −70 °C until use. As stated in the product information sheet: (1) Polymerase chain reaction protocol was used for pathogenicity testing of all liver specimens (male human donners of mixed age and different health states). (2) Each liver was tested to be negative for hepatitis B and C, HIV1 & 2 and HTLV1 & 2 negative. (3) The protein content was 20 mg/mL in 250 mM sucrose to keep HLMs active. Total cytochrome P450 enzymes and FMO activities are mentioned in the product information sheet. Organic solvents are of HPLC grade, and reference powders are of analytical grade. HPLC grade water was prepared inhouse by a Milli-Q plus filtration instrument that was purchased from Millipore company (Burlington, MA, USA). Tepotinib (99.87 %) and NADPH (99.99%) were procured from MedChem. Express Company (Monmouth, NJ, USA). Acetonitrile, formic acid (HCOOH), ammonium formate (NH_4_COOH) and potassium cyanide (KCN) were procured from Sigma-Aldrich company (St. Louis, MO, USA).

### 3.2. Chromatography Conditions

Agilent 1200 rapid resolution liquid chromatography (RRLC) coupled to an Agilent 6410 triple-quadrupole with an electrospray ionization source was used for chromatographic separation and mass spectrometric studies for TEP-related metabolites (Agilent, Santa Clara, CA, USA). The chromatographic conditions and mass spectrometric parameters were optimized for TEP, its related metabolites and cyano adducts to attain good separation with mass sensitivity. The chromatography was performed on a reversed phase C_18_ column (length: 150 mm; internal diameter (ID): 2.1 mm; particle size: 3.5 µm) at 23 ± 2 °C. The aqueous part pH of the mobile phase (5 mM ammonium formate) was fixed at 3.5 at 0.3 mL/min flow rate. A binary system was used for separation of incubation matrix components that consisted of solvent A which is aqueous and solvent B which is ACN. The stepwise gradient was 5% B (0–5 min), 5 to 60% B (5–40 min), 60 to 90% B (40–60 min) and 90 to 5% B (60–65 min). The post-time was 15 min. The injection volume of samples was 15 µL with a total run time of 65 min.

Fragment ions for TEP, its related metabolites and cyano adducts were formed inside the collision cell by collision-induced dissociation (CID) using nitrogen gas as a collision gas. Nitrogen gas (flow rate: 11 L/min and pressure: 55 psi) was also used as a drying gas in the electrospray ionization source (ESI) that was generated by a nitrogen generator. ESI operated in the positive charge ionization mode was utilized for ion generation. Flow injection analysis was used for mass spectrometric parameter optimization to attain the highest ion intensity. The values of capillary voltage and ESI temperature were adjusted at 4000 V and 350 °C, respectively. Data acquisition was controlled using the Mass Hunter software. Characterization of TEP metabolites was performed using product ion mode (PI) for the mass transitions (precursor to fragment ions). The fragmentor voltage was adjusted at 145 V with collision energy at 25 eV for TEP metabolites. PI mode was used for structural identification to remove any interference from the HLM matrix components and increase the LC-MS/MS method sensitivity.

### 3.3. In Silico Prediction of TEP Metabolism Using WhichP450^TM^ Module of StarDrop Software

Identification of the vulnerable metabolic sites of TEP chemical structure was performed by running the WhichP450™ module after uploading the chemical structure in SMILE format. Each site vulnerability of metabolism was indicated by the composite site lability (CSL). The regioselectivity of metabolism by the major isoforms was also proposed. The results are shown in the form of a pie chart that was used for indication of the most likely CYP450 isoform that has a major role in TEP metabolism [29].

### 3.4. In Silico Prediction of the Toxicity of TEP Metabolites and Reactivity Using DEREK Software and Xenosite Reactivity Model

Screening for the structural alerts of TEP metabolites was performed the using DEREK module of StarDrop software. Structural modifications were also proposed at the bioactive centers that stop the bioactivation process using freely available Xenosite reactivity by uploading the SMILES of the TEP chemical structure [30].

### 3.5. HLM Incubation

The detection of TEP-formed metabolites was performed by incubation of 30 µM TEP in 1 mL 50 mM phosphate buffer (pH 7.4) that contained 1.0 mg/mL HLMs and 3.3 mM MgCl_2_ in a shaking water bath (37 °C for 2 h). The in vitro TEP metabolism was initiated by adding NADPH (1.0 mM) as a cofactor and stopped by adding acetonitrile (2 mL) as a precipitating agent. The precipitates were removed by centrifugation (9000× *g* for 15 min at 4 °C). The purified supernatants were transferred to clean tubes for evaporation to dryness under a stream of nitrogen gas. The residues were reconstituted in mobile phase and then loaded in HPLC vials. Fifteen microliters of sample was analyzed using LC-MS/MS [34,35,36,37]. For checking reactive iminium intermediates, the same experimental procedure mentioned above was repeated plus the addition of 100 µL of 1 mM KCN (0.1 mM in the final incubation) before the addition of NADPH.

### 3.6. Identification of TEP-Reactive Metabolites

After full MS scan (*m*/*z* 50 to 600), the extraction of selected MIP was utilized to locate predicted metabolites (in silico outcomes) in the TIC. Molecular ions were subjected to fragmentation inside the collision cell by the collision-induced dissociation (CID) technique to generate fragment ions (FI), which were used to reconstruct the chemical structures of the original MI. Thus, the fragment ions pattern of TEP was used to guide the confirmation and interpretation of the proposed chemical structures of the in vitro metabolites and cyano adducts generated in TEP metabolism. The TEP HLM incubations were repeated in the presence of potassium cyanide (KCN) for capturing bioactive intermediates. To confirm the outcomes, all experiments were repeated three times using controls (without HLMs or NADPH).

## 4. Conclusions

Three metabolic pathways including oxidation, hydroxylation and *N*-demethylation resulted in the formation of four in vitro phase I TEP metabolites. The bioactivation sequence of TEP resulted in the generation of four cyano adducts. The TEP chemical structure contains the cyclic tertiary amine ring (piperidine ring) that was bioactivated because of the generation of iminium ions (reactive intermediates), which could covalently bind to DNA or other important hormones and proteins inside the human body. The TEP bioactivation mechanism was revealed using LC-MS/MS. These outcomes facilitate further work on reducing TEP toxicity by synthesis of new drug series that are characterized by an increased safety profile and at the same time maintaining their pharmacological action. Blocking or replacing isosteric substituents to the bioactive carbon at the piperidine ring moiety would likely disturb or block hydroxylation reaction, which would stop TEP bioactivation steps. In silico toxicological assessment of TEP and its related metabolites was performed using the DEREK module of StarDrop software, which revealed proposed side effects and structural alerts of TEP. Phospholipidosis was stopped as predicted by the DEREK module of StarDrop software utilizing small structural modification.

## Figures and Tables

**Table 1:** Qualitative toxicity prediction of the TEP and its phase I metabolites by DEREK module of StarDrop software.

TEP and Its Phase I Metabolites	Phospholipidosis	Skin Sensitization	Nephrotoxicity	Chromosome Damage, Teratogenicity, Carcinogencity, Mutagenicity and Genotoxicity
	Piperidine	Hydrazine	Halogenated nitrile	
TEP	Plausible	Equivocal	Equivocal	NA
M1	NA *	Plausible	Equivocal	NA
M2	NA	Equivocal	Equivocal	NA
M3	NA	Equivocal	Equivocal	NA
M4	NA	Equivocal	Equivocal	NA

* NA: not applicable.

**Table 2:** In vitro phase-I metabolites and cyano adducts of TEP.

	MS Scan	Fragment Ions	Elution Time (min)	Metabolic and Bioactivation Pathways
TEP	493	112	25.1	Main drug
**Phase-I Metabolites**				
M1	493	382, 296, 185, 112, 72	26.6	*N*-demethylation and α-oxidation at piperidine ring
M2	479	382, 185, 98	24.6	*N*-demethylation at piperidine ring
M3	507	310, 126	28.3	α-Oxidation at piperidine ring
M4	509	128	28.4	α-Hydroxylation at piperidine ring
**Reactive Metabolites**				
TEPCN518	518	491, 459, 137, 110, 104	39.2	Cyano attack at bioactivated α-carbon of piperidine ring
TEPCN520	520	493, 475, 278, 139	42.9	*N*-demethylation, hydroxylation and cyano attack at bioactivated α-carbon of piperidine ring
TEPCN534a	534	516, 475, 296, 153	28.9	Hydroxylation and cyano addition at α-carbon of piperidine ring
TEPCN534b	534	491, 382, 210, 153, 110		Hydroxylation at methylene carbon attached to piperidine ring and cyano attack at bioactivated α-carbon of piperidine ring
